# Momentary Self-esteem as a Process Underlying the Association Between Childhood Trauma and Psychosis: Experience Sampling Study

**DOI:** 10.2196/34147

**Published:** 2023-04-05

**Authors:** Maud Daemen, Therese van Amelsvoort, GROUP investigators, Ulrich Reininghaus

**Affiliations:** 1 Department of Psychiatry and Neuropsychology School for Mental Health and Neuroscience Maastricht University Maastricht Netherlands; 2 Department of Public Mental Health, Central Institute of Mental Health Medical Faculty Mannheim University of Heidelberg Mannheim Germany; 3 See Acknowledgments; 4 Centre for Epidemiology and Public Health, Health Service and Population Research Department, Institute of Psychiatry, Psychology & Neuroscience King’s College London London United Kingdom

**Keywords:** psychosis, self-esteem, childhood trauma, childhood adversity, experience sampling method, ecological momentary assessment

## Abstract

**Background:**

Exposure to childhood trauma is associated with an increased risk of developing and maintaining psychotic symptoms later in life. Self-esteem might be an important psychological process underlying the association between childhood trauma and psychosis, but there is only limited evidence to support this claim, especially in daily life.

**Objective:**

In this study, we aimed to investigate whether exposure to childhood trauma (physical, emotional, and sexual abuse and physical and emotional neglect) moderates the cross-sectional and temporal associations between self-esteem and psychotic experiences in patients with psychotic disorders, their first-degree relatives, and controls.

**Methods:**

We assessed momentary self-esteem and psychotic experiences in daily life using the experience sampling method in 139 patients with psychotic disorders, 118 first-degree relatives of patients with psychotic disorders, and 111 controls. Childhood trauma was measured using the Childhood Trauma Questionnaire. We fitted linear mixed models and added two-way and three-way interaction terms to test the hypotheses.

**Results:**

The association between momentary self-esteem and psychotic experiences in daily life was modified by prior exposure to high versus low levels of several types of childhood trauma, that is, physical (*χ*^2^_2_=24.9, family-wise error-corrected *P*<.001) and sexual abuse (*χ*^2^_2_=15.9, *P*<.001) and physical neglect (*χ*^2^_2_=116.7, *P*<.001). Specifically, momentary self-esteem was associated with more intense psychotic experiences in patients exposed to high versus low levels of physical neglect, in relatives exposed to high versus low levels of physical abuse, and in relatives and controls exposed to high versus low levels of sexual abuse. When investigating temporal order, the results showed no evidence that childhood trauma modified the temporal associations between self-esteem at t_n-1_ and psychotic experiences at t_n_ or those between psychotic experiences at t_n-1_ and self-esteem at t_n_.

**Conclusions:**

The association between self-esteem and psychotic experiences in daily life was found to be stronger in those exposed to high versus low levels of several types of childhood trauma (ie, physical abuse, sexual abuse, and physical neglect).

## Introduction

### Background

Exposure to childhood trauma can have persistent adverse effects on an individual’s well-being, social development, and physical and mental health [1]. Childhood trauma refers to potentially harmful experiences of physical, emotional, and sexual abuse and physical and emotional neglect during childhood [2]. Accumulating evidence suggests that childhood trauma is associated with psychotic disorders [3-6] and increases the risk of developing and maintaining psychotic symptoms later in life [3,7,8]. The study by Norman et al [9] showed that the prevalence of childhood trauma in patients with psychotic disorders is very common; 82 of their 100 participants (patients receiving treatment from an early psychosis clinic) reported exposure to childhood trauma. Similarly, other studies have shown that patients with psychotic disorders reported having experienced disproportionate levels of childhood trauma [3,7,8,10-12]. Furthermore, the results of the meta-analysis conducted by Varese et al [3] suggested that 1 out of 3 patients might not have developed psychosis if adversity were to be eliminated as a risk factor (assuming causality), which indicates that childhood trauma plays a prominent role in the development of psychosis. It has also been demonstrated that psychotic symptoms are more severe in patients exposed to childhood trauma [9,13]. A prospective cohort study indicated that the incidence of psychotic experiences decreased significantly when exposure to trauma ceased over the course of the study [14]. Finally, a systematic review revealed that exposure to childhood adversity was associated with the persistence of psychotic symptoms in both the general population and clinical studies [15], suggesting that childhood adversity may play an important role in the maintenance of psychotic symptoms in patients with enduring psychotic disorders.

Evidence for familial liability to psychosis [16-18] shows that first-degree relatives of patients with psychosis have an increased risk of developing psychotic disorders [17,19]. In addition, although exposure to childhood trauma is higher in patients with psychotic disorders, patients and their siblings share a degree of exposure to childhood trauma [20], as they also share many sociodemographic, parental, and developmental characteristics [21].

Several putative psychological mechanisms and processes have been posited to be involved in the association between childhood trauma and psychosis [22,23]. Self-esteem is one such process. A systematic review indicated that exposure to childhood trauma may contribute to low self-esteem [24]. Low self-esteem has also been found to be common in patients with psychotic disorders [25-28]. Moreover, self-esteem has been shown to be involved in the development and maintenance of psychotic symptoms [28-32]. Lower levels of momentary self-esteem have been found to be associated with an increased intensity of psychotic experiences in daily life [33]. Prior exposure to childhood trauma may affect cognition [34], which contributes to the development of a complex system consisting of negative views of one’s self, the world, and the future [35]. Some researchers have suggested that self-esteem is involved in the pathway from negative life events (eg, childhood trauma) to psychosis, but evidence to support this claim remains limited [29,36,37]. A few studies have investigated self-esteem and closely related processes such as negative self-schemas, or negative beliefs about self and others, as an underlying process in the association between childhood trauma and psychotic experiences [38-42]. In contrast, other researchers have suggested low self-esteem to be a product of an individual’s experience of psychosis, for example, owing to hospitalization, the loss of their social role or rank, or stigmatization [43,44].

Self-esteem and psychotic experiences are often assessed using cross-sectional measures, with global scores [38-41]. Another way of collecting data on these variables is through the experience sampling method (ESM). ESM assesses moment-to-moment variations in thoughts, feelings, and behaviors in daily life. It generates longitudinal data with a very limited recall bias and high ecological validity [45].

### Objective

Overall, there is limited evidence that exposure to childhood trauma moderates the association between self-esteem and psychotic experiences, especially in daily life. Exposure to childhood trauma plays an important role in the development and maintenance of psychosis [17-19,46], but unfortunately, preventing childhood trauma remains challenging. By investigating whether self-esteem is a relevant underlying process in the association between childhood trauma and psychotic experiences, we might be able to identify individuals exposed to (high levels of) childhood trauma in the early stages and target self-esteem to prevent a full-blown psychotic disorder.

Therefore, we aimed to investigate whether cross-sectional and temporal associations of momentary self-esteem and psychotic experiences in daily life were modified by prior exposure to childhood trauma (ie, physical, emotional, and sexual abuse and physical and emotional neglect) in patients with enduring psychotic disorders, their first-degree relatives, and controls. Specifically, using data from the Genetic Risk and Outcome in Psychosis (GROUP) study [47], a large multicenter study of patients with psychotic disorders, their first-degree relatives, and controls without a family history of psychotic disorders, we aimed to test the following hypotheses:

1. In patients, their first-degree relatives, and controls, the magnitude of associations of self-esteem and psychotic experiences in daily life (both measured with the ESM) is stronger in individuals exposed to high levels of each type of childhood trauma (ie, physical, emotional, and sexual abuse and physical and emotional neglect) than in those exposed to low levels of childhood trauma (measured using the Childhood Trauma Questionnaire [CTQ]; hypothesis 1).
2. The difference in the magnitude of associations of self-esteem and psychotic experiences in daily life between those exposed to high levels of childhood trauma and those exposed to low levels of childhood trauma is (1) greater in patients than in controls, (2) greater in relatives than in controls, and (3) greater in patients than in relatives (hypothesis 2).
3. In patients, their first-degree relatives, and controls, the temporal association (1) between self-esteem at t_n-1_ and psychotic experiences at t_n_ and (2) between psychotic experiences at t_n-1_ and self-esteem at t_n_ in daily life (both measured with the ESM) is stronger in individuals exposed to high levels of each type of childhood trauma than those exposed to low levels of childhood trauma (measured using the CTQ; hypothesis 3).
4. The difference in the magnitude of temporal associations of (1) self-esteem and psychotic experiences and (2) psychotic experiences and self-esteem in daily life between those exposed to high levels of childhood trauma and those exposed to low levels of childhood trauma is (1) greater in patients than in controls, (2) greater in relatives than in controls, and (3) greater in patients than in relatives (hypothesis 4).

## Methods

### Sample

A sample of patients with psychotic disorders, their first-degree relatives, and controls without a family history of psychotic disorders was recruited in the GROUP study [47], a large longitudinal multicenter study in the Netherlands and Belgium. Individuals with psychotic disorders were recruited from regional psychosis care facilities or academic centers in selected geographical areas. The patients’ relatives were contacted after obtaining written informed consent. Participants in the control group were recruited by contacting random addresses in the same geographical areas as the patients. To be eligible, participants had to be between 16 and 50 years old and their command of Dutch language had to be sufficient. As an additional criterion, patients had to be diagnosed with a nonaffective psychotic disorder according to the Diagnostic and Statistical Manual of Mental Disorders, Fourth Edition criteria [48]. In the control group, individuals with a family history of psychotic disorders were excluded.

### Ethics Approval

Ethics approval was provided by the Ethical Review Board of the University Medical Centre Utrecht (METC: 0 4/003- O), and all participants gave written informed consent. For this analyses, only participants who completed the CTQ and, in line with previous ESM studies [49], at least one-third (33.3%) of the ESM assessments were included.

### Data Collection

#### Basic Sample Characteristics

Using a sociodemographic schedule, data on age, gender, ethnicity, marital status, and educational level (adapted from the Dutch Standard Classification of Education) [50] were collected.

#### Childhood Trauma

The Dutch version of the 25-item CTQ-short version [2] was used to assess childhood trauma at the baseline. The 25 items inquire about 5 types of trauma in childhood (emotional, physical, and sexual abuse and emotional and physical neglect). All 5 types of trauma are covered with 5 items rated on a 5-point Likert scale (1=never true to 5=very often true). The CTQ–short version has shown to be reliable and to provide adequate content coverage. There is also good evidence of criterion validity in both adolescent patients with a psychiatric disorder and individuals from a normative community sample [2]. For the analyses, the mean score for each type of trauma was used.

#### ESM Procedure

The ESM was used at the 6-year follow-up assessment to collect data on self-esteem and psychotic experiences in daily life. Participants were offered an ESM briefing session that provided detailed instructions on the ESM procedure. Participants received a dedicated digital device (ie, PsyMate, ECS International B.V.), which emitted a beep at 10 semirandom times a day, within 90-minute time blocks, between 7:30 AM and 10:30 PM for 6 consecutive days. Each time the PsyMate emitted a beep, the participants were asked to complete an ESM questionnaire immediately after the beep. A debriefing session was scheduled after 6 days. Research has shown that the ESM in samples of patients with psychosis and their relatives is feasible, reliable, and valid [51-54].

#### ESM Measures

To assess momentary self-esteem, the mean score of the following 2 ESM items was used: “I like myself” and “I doubt myself” (reversed) [55] (following the approach by Geldhof et al [56] to analyze multilevel reliability; within-person *α*=.22; between-person *α*=.76). To assess the intensity of psychotic experiences, the mean score of the following 8 ESM items was used: “My thoughts are influenced by others,” “I can’t get these thoughts out of my head,” “I feel unreal,” “My thoughts can’t be expressed in words,” “I feel suspicious,” “I hear voices,” “I see things that aren’t really there,” and “I am afraid I will lose control” [57,58] (within-person *α*=.64; between-person *α*=.85). All the items were measured on a 7-point Likert scale, ranging from 1 (“not at all”) to 7 (“very much”).

### Statistical Analysis

For this analyses, we used release 7.0 of the overall GROUP database and release 2.0 of the ESM data. The “mixed” command in Stata 13.0 (StataCorp) was used to fit linear mixed models. Because ESM data include multiple observations within each participant, they were treated as nested within participants and participants nested within families. Restricted maximum likelihood estimation was used to fit the models and estimated the associations between momentary self-esteem as the continuous independent variable and psychotic experiences as the outcome variable. We controlled for the potential confounders of age, gender, ethnicity (people of color or White), level of education, and marital status.

We then added two-way (self-esteem × abuse, self-esteem × group, and abuse × group) and three-way (self-esteem × abuse × group) interaction terms to test whether associations between self-esteem and psychotic experiences were modified by prior exposure to high (mean + 1 SD of continuous CTQ scores) versus low levels (mean − 1 SD of continuous CTQ scores) [59,60] of each type of childhood abuse to each group (patients, relatives, and controls). We standardized the continuous ESM and CTQ variables (mean 0, SD 1) to interpret significant three-way interaction terms [61]. Wald tests were used to test the hypothesis that the interaction effects were equal to zero. For the three-way interaction models, the significance levels of the Wald tests were adjusted to correct for type-I errors using family-wise error-corrected *P* value, which were computed by multiplying the unadjusted *P* value by the total number of tests. The “lincom” command was used to compute linear combinations of coefficients to test hypotheses 1 and 2. Next, we conducted time-lagged analyses to test hypotheses 3 and 4. To do so, we generated within-subject lagged variables of self-esteem and psychotic experiences (at t_n-1_ and t_n_) and fitted linear mixed models.

## Results

### Basic Characteristics

The third-wave (6-year follow-up) data of the GROUP study was completed by 486 participants (patients: n=194, 39.9%; relatives: n=169, 34.8%; and controls: n=123, 25.3%). Participants who did not complete the CTQ in the first wave (30/486, 6.2%) and a minimum of one-third of the ESM assessments in the third wave (88/486, 18.1%) were excluded from the analysis, resulting in a sample of 368 participants (patients: n=139, 37.8%; relatives: n=118, 32.1%; and controls: n=111, 30.2%). The basic characteristics of the excluded participants of the third-wave sample were broadly similar to those of the included participants (Multimedia Appendix 1). Overall, the basic characteristics of the included patients, their relatives, and controls were similar, except that included participants of all 3 groups were more often White compared with the excluded participants. Compared with the relatives and controls, the patients were younger and had a lower educational level. The patient group consisted of more men and were more often unmarried compared with the other 2 groups (Table 1). Patients reported higher levels of all types of childhood trauma (emotional abuse, *P*<.001; physical abuse, *P*=.02; sexual abuse, *P*=.02; emotional neglect, *P*=.01; physical neglect, *P*=.01) than the controls (Table 2). Patients also reported higher levels of emotional abuse (adjusted *β*=.58, 95% CI 0.35-0.80; *P<*.001), physical abuse (adjusted *β*=.25, 95% CI 0.06-0.44; *P=*.01), and sexual abuse (adjusted *β*=.41, 95% CI 0.20-0.63; *P*<.001) than their first-degree relatives, where adjusted *β* are standardized regression coefficients. Although patients reported higher levels of emotional and physical neglect, the differences between the patients and relatives were not statistically significant (patients: adjusted *β*=.22, 95% CI −0.02 to 0.46; *P=*.07 and relatives: adjusted *β*=.08, 95% CI −0.13 to 0.29; *P=*.47; Table 3). The levels of all types of childhood trauma were similar in controls and relatives.

**Table 1:** Basic characteristics.

			Patients (n=139)		Relatives (n=118)		Controls (n=111)		Test statistics							*P* value
									*F* test (*df*)			*χ*^2^ (*df*)				
Age (years), mean (SD)			34.5 (8.3)		35.5 (8.7)		41.0 (11.5)		15.87 (2)			N/A^a^			<.001	
**Gender (patients: n=135; relatives: n=118; control: n=111), n (%)^b^**										N/A			34.8 (2)			<.001
	Men	88 (65.2)		47 (39.8)		33 (29.7)										
	Women	47 (34.8)		71 (60.2)		78 (70.3)										
**Ethnicity (patients: n=139; relatives: n=118; control: n=111), n (%)**										N/A			5.2 (2)			.08
	White	123 (88.5)		107 (90.7)		107 (96.4)										
	People of color	16 (11.5)		11 (9.3)		4 (3.6)										
**Level of education (patients: n=139; relatives: n=118; control: n=111), n (%)**										N/A			42.2 (4)			<.001
	Low	35 (25.2)		8 (6.8)		3 (2.7)										
	Middle	60 (43.2)		45 (38.1)		42 (37.8)										
	High	44 (31.6)		65 (55.1)		66 (59.5)										
**Marital status (patients: n=139; relatives: n=118; control: n=93), n (%)^c^**										N/A			71.1 (4)			<.001
	Not married	101 (72.7)		37 (31.4)		25 (26.9)										
	Married or living together	28 (20.1)		76 (64.4)		62 (66.7)										
	Divorced	10 (7.2)		5 (4.2)		6 (6.4)										
Current antipsychotic use (patients: n=100), n (%)^d^			98 (98)		N/A		N/A		N/A			N/A			N/A	
**Childhood trauma, mean (SD)**																
	Emotional abuse^e^	9.5 (4.2)		7.5 (3.1)		7.1 (3.4)		15.51 (2)			N/A			<.001		
	Physical abuse^e^	6.6 (2.9)		5.7 (2.2)		5.5 (1.6)		8.53 (2)			N/A			<.001		
	Sexual abuse^e^	6.3 (3.1)		5.6 (2.6)		5.9 (3.0)		1.93 (2)			N/A			.15		
	Emotional neglect^f^	11.4 (4.1)		10.6 (4.1)		9.2 (3.9)		9.25 (2)			N/A			<.001		
	Physical neglect	7.2 (2.4)		6.6 (2.5)		6.0 (1.9)		9.16 (2)			N/A			<.001		

^a^N/A: not applicable.

^b^Missing values: 4/368, 0.01%.

^c^Missing values: 18/368, 4.9%.

^d^Missing values: 39/139, 28.1%.

^e^Missing values: 2/368, 0.5%.

^f^Missing values: 3/368, 0.8%.

**Table 2:** Categorical Childhood Trauma Questionnaire severity scores by group.

		Patients (n=139), n (%)	Relatives (n=118), n (%)	Controls (n=111), n (%)	*χ*^2^ (*df*)		*P* value	
**Emotional abuse^a^ (patients: n=139; relatives: n=118; control: n=109)**						32.8 (6)		<.001
	Severe to extreme	13 (9.3)	4 (3.4)	3 (2.8)				
	Moderate to severe	19 (13.7)	2 (1.7)	6 (5.5)				
	Mild to moderate	34 (24.5)	22 (18.6)	13 (11.9)				
	None	73 (52.5)	90 (76.3)	87 (79.8)				
**Physical abuse^a^ (patients: n=138; relatives: n=118; control: n=110)**						15.5 (6)		.02
	Severe to extreme	8 (5.8)	3 (2.5)	3 (2.7)				
	Moderate to severe	11 (8)	2 (1.7)	1 (0.9)				
	Mild to moderate	7 (5.1)	5 (4.3)	2 (1.8)				
	None	112 (81.1)	108 (91.5)	104 (94.6)				
**Sexual abuse^a^ (patients: n=139; relatives: n=118; control: n=109)**						15.5 (6)		.02
	Severe to extreme	9 (6.5)	4 (3.4)	5 (4.6)				
	Moderate to severe	14 (10.0)	2 (1.7)	6 (5.5)				
	Mild to moderate	19 (13.7)	11 (9.3)	6 (5.5)				
	None	97 (69.8)	101 (85.6)	92 (84.4)				
**Emotional neglect^b^ (patients: n=138; relatives: n=118; control: n=109)**						17.6 (6)		.01
	Severe to extreme	10 (7.2)	9 (7.6)	6 (5.5)				
	Moderate to severe	20 (14.5)	11 (9.3)	4 (3.7)				
	Mild to moderate	60 (43.5)	44 (37.3)	35 (32.1)				
	None	48 (34.8)	54 (45.8)	64 (58.7)				
**Physical neglect (patients: n=139; relatives: n=118; control: n=111)**						15.9 (6)		.01
	Severe to extreme	6 (4.3)	6 (5.1)	2 (1.8)				
	Moderate to severe	18 (13.0)	6 (5.1)	4 (3.6)				
	Mild to moderate	21 (15.1)	13 (11.0)	9 (8.1)				
	None	94 (67.6)	93 (78.8)	96 (86.5)				

^a^Missing values: 2/368, 0.5%.

^b^Missing values: 3/268, 0.8%.

**Table 3:** Differences in scores of Childhood Trauma Questionnaire severity between groups.

	Patients versus controls			Relatives versus controls			Patients versus relatives	
	Adjusted *β* (95% CI)^a^	*P* value	Adjusted *β* (95% CI)		*P* value	Adjusted *β* (95% CI)		*P* value
Emotional abuse	.67 (0.42 to 0.92)	<.001	.09 (−0.14 to 0.32)		.44	.58 (0.35 to 0.80)		<.001
Physical abuse	.30 (0.09 to 0.51)	.005	.05 (−0.14 to 0.25)		.58	.25 (0.06 to 0.44)		.01
Sexual abuse	.42 (0.19 to 0.66)	<.001	.01 (−0.21 to 0.23)		.92	.41 (0.20 to 0.63)		<.001
Emotional neglect	.51 (0.25 to 0.78)	<.001	.30 (0.05 to 0.54)		.02	.22 (−0.02 to 0.46)		.07
Physical neglect	.26 (0.03 to 0.50)	.03	.19 (−0.02 to 0.40)		.08	.08 (−0.13 to 0.29)		.47

^a^CI adjusted for age, gender, ethnicity, education, and marital status.

### Cross-sectional Association Between Self-esteem and Psychotic Experiences by Childhood Trauma and Group

As shown in Table 4, after controlling for age, gender, ethnicity, education, and marital status, we found strong evidence that the association between self-esteem and psychotic experiences was modified by exposure to childhood trauma, as indicated by the statistically significant interaction effect of self-esteem × CTQ total score × group (*χ*^2^_2_=9.0, *P*=.01). We found statistically significant interaction effects (all *P*≤.001) of self-esteem × physical abuse × group, self-esteem × sexual abuse × group, and self-esteem × physical neglect × group. However, there was no evidence of interaction effects of self-esteem × emotional abuse × group (*χ*^2^_2_=5.4, *P*=.07) and self-esteem × emotional neglect × group (*χ*^2^_2_=1.8, *P*=.41).

**Table 4:** Associations between momentary self-esteem and psychotic experiences by childhood trauma in patients, relatives, and controls^a^.

			Outcome: psychotic experiences												
			Patients			Relatives			Controls			Wald test for interaction			
			Adjusted *β*^b^ (95% CI)	*P* value	Adjusted *β* (95% CI)		*P* value	Adjusted *β* (95% CI)		*P* value	*χ*^2^ (*df*)			*P* value^c^	
**Momentary SE^d^ × childhood trauma × group^e^**													9.0 (2)		.01
	**Level of childhood trauma**														
		High (mean + 1 SD)	−.20 (−0.25 to −0.15)	<.001	−.29 (−0.32 to −0.27)		<.001	−.15 (−0.19 to −0.12)		<.001					
		Average (mean)	−.17 (−0.20 to −0.14)	<.001	−.27 (−0.29 to −0.25)		<.001	−.18 (−0.20 to −0.15)		<.001					
		Low (mean − 1 SD)	−.14 (−0.18 to −0.10)	<.001	−.25 (−0.28 to −0.22)		<.001	−.20 (−0.24 to −0.16)		<.001					
		High versus low	−.06 (−0.13 to 0.01)	.12	−.04 (−0.08 to −0.01)		.03	.05 (−0.00 to 0.10)		.07					
Momentary SE × emotional abuse × group^e^													5.4 (2)		.07
**Momentary SE × physical abuse × group^e^**													24.9 (2)		<.001
	**Level of physical abuse**														
		High (mean + 1 SD)	−.16 (−0.22 to −0.10)	<.001	−.32 (−0.35 to −0.30)		<.001	−.08 (−0.12 to −0.04)		<.001					
		Average (mean)	−.16 (−0.18 to −0.13)	<.001	−.26 (−0.29 to −0.24)		<.001	−.11 (−0.14 to −0.08)		<.001					
		Low (mean − 1 SD)	−.16 (−0.21 to −0.11)	<.001	−.21 (−0.24 to −0.17)		<.001	−.14 (−0.18 to −0.09)		<.001					
		High versus low	0 (−0.09 to 0.09)	.99	−.12 (−0.15 to −0.08)		<.001	.06 (−0.00 to 0.12)		.058					
**Momentary SE × sexual abuse × group^e^**													15.9 (2)		<.001
	**Level of sexual abuse**														
		High (mean + 1 SD)	−.11 (−0.17 to −0.06)	<.001	−.29 (−0.32 to −0.27)		<.001	−.08 (−0.12 to −0.04)		<.001					
		Average (mean)	−.15 (−0.18 to −0.13)	<.001	−.27 (−0.29 to −0.25)		<.001	−.11 (−0.14 to −0.08)		<.001					
		Low (mean − 1 SD)	−.19 (−0.24 to −0.15)	<.001	−.25 (−0.28 to −0.22)		<.001	−.13 (−0.17 to −0.09)		<.001					
		High versus low	.08 (−0.01 to 0.16)	.07	−.05 (−0.08 to −0.02)		.001	.05 (0.00 to 0.10)		.049					
Momentary SE × emotional neglect × group													1.8 (2)		.41
**Momentary SE × physical neglect × group^e^**													116.7 (2)		<.001
	**Level of physical neglect**														
		High (mean + 1 SD)	−.23 (−0.28 to −0.19)	<.001	−.29 (−0.32 to −0.27)		<.001	−.08 (−0.12 to −0.04)		<.001					
		Average (mean)	−.17 (−0.20 to −0.14)	<.001	−.28 (−0.30 to −0.25)		<.001	−.11 (−0.14 to −0.08)		<.001					
		Low (mean − 1 SD)	−.11 (−0.14 to −0.07)	<.001	−.26 (−0.29 to −0.23)		<.001	−.13 (−0.17 to −0.09)		<.001					
		High versus low	−.13 (−0.19 to −0.07)	<.001	−.03 (−0.07 to 0.01)		.14	.05 (−0.01 to 0.11)		.11					

^a^Adjusted for age, gender, ethnicity, level of education, and marital status.

^b^Adjusted *β*: standardized regression coefficients (continuous independent variables were standardized [mean 0, SD 1] for interpreting significant three-way interaction terms and examining the difference in associations between high [mean + 1 SD], average [mean], and low [mean − 1 SD] levels of childhood trauma within and across groups [patients, relatives, and controls]).

^c^Family-wise error-corrected *P* values were computed by multiplying the unadjusted *P* value by the total number of tests to adjust the significance levels of Wald tests for three-way interactions.

^d^SE: self-esteem.

^e^Three-way interaction included in the following model (with y_ij_ psychotic experiences as the outcome variable): y_ij_ = *β*_0_ + *β*_1_(self-esteem) + *β*_2_(childhood trauma_j_) + *β*_3_(group_j_) + *β*_4_(self-esteem_ij_ × childhood trauma_j_) + *β*_5_(self-esteem_ij_ × group_j_) + *β*_6_(childhood trauma_j_ × group_j_) + *β*_7_(self-esteem_ij_ × childhood trauma_j_ × group_j_) + *ε*_ij_ (full model not shown and available upon request).

### Within-Group Comparison—Hypothesis 1

Lower levels of momentary self-esteem were associated with more intense psychotic experiences in relatives exposed to high levels of childhood trauma in general compared with those exposed to low levels of childhood trauma (adjusted *β_high vs low_*=−.04; *P*=.03). There was no evidence that this association was stronger in patients or controls exposed to high versus low levels of childhood trauma in general. Specifically, in relatives, we found a stronger association between lower self-esteem and more intense psychotic experiences for those exposed to high versus low levels of physical abuse (adjusted *β_high vs low_*=−.12; *P*<.001); however, we did not find evidence that this association was modified in patients or controls (Figure S1 in Multimedia Appendix 2). Furthermore, lower self-esteem was associated with more intense psychotic experiences in relatives and controls exposed to high versus low levels of sexual abuse (relatives: adjusted *β_high vs low_*=−.05; *P*<.001 and controls: adjusted *β_high vs low_*=.05; *P*=.049) but not in patients (Figure S2 in Multimedia Appendix 2). Finally, in patients, we found a stronger association between lower self-esteem and more intense psychotic experiences in those exposed to high versus low levels of physical neglect (adjusted *β_high vs low_*=−.13; *P*<.001; Figure S3 in Multimedia Appendix 2). However, there was no evidence that this association was modified in relatives and controls.

### Between-Group Comparison—Hypothesis 2

Next, we examined the differences in the magnitude of associations of self-esteem and psychotic experiences between those exposed to high versus low levels of childhood trauma in general, physical and sexual abuse, and physical neglect across groups. These differences in magnitude were only examined if both groups showed significant (*P*<.05) within-group associations (Table 5). When comparing relatives with controls, we found differences in the magnitude of associations between self-esteem and psychotic experiences between those exposed to high versus low levels of sexual abuse (adjusted *β_high vs low_*=−.10; *P*<.001), with differences in magnitude of association being greater in relatives.

**Table 5:** Difference in associations in those exposed to high versus low levels of childhood trauma across groups (Δ high vs low).

			Outcome: psychotic experiences											
			Patients versus controls				Relatives versus controls					Patients versus relatives		
			Adjusted *β* (95% CI)		*P* value		Adjusted *β* (95% CI)		*P* value		Adjusted *β* (95% CI)			*P* value
**Self-esteem**														
	Δ High versus low childhood trauma across groups	N/A^a^		N/A		N/A		N/A		N/A			N/A	
	Δ High versus low physical abuse across groups	N/A		N/A		N/A		N/A		N/A			N/A	
	Δ High versus low sexual abuse across groups	N/A		N/A		−.10 (−0.16 to −0.04)		<.001		N/A			N/A	
	Δ High versus low physical neglect across groups	N/A		N/A		N/A		N/A		N/A			N/A	

^a^N/A: not applicable.

### Temporal Association Between Self-esteem and Psychotic Experiences by Childhood Trauma and Group

Findings regarding the temporal associations between momentary self-esteem and psychotic experiences in patients, relatives, and controls are shown in Tables 6 and 7. After controlling for age, gender, ethnicity, education, and marital status, we found no evidence that associations between self-esteem at t_n-1_ and psychotic experiences at t_n_ or between psychotic experiences at t_n-1_ and self-esteem at t_n_ were modified by exposure to any of the types of childhood trauma (ie, emotional, physical, and sexual abuse and emotional and physical neglect) within (hypothesis 3) and across (hypothesis 4) groups (ie, patients, relatives, and controls).

**Table 6:** Effect of self-esteem and psychotic symptoms at t_n-1_ on psychotic experiences at t_n_ by group and modified by childhood trauma^a^.

				Patients				Relatives					Controls					Wald test for interaction			
				Adjusted *β*^b^ (95% CI)		*P* value		Adjusted *β* (95% CI)		*P* value		Adjusted *β* (95% CI)			*P* value		*χ*^2^ (*df*)			*P* value^c^	
**Self-esteem t_n−1_**																					
	**Emotional abuse**																	0.07 (2)			.80
		High (mean + 1 SD)	−.04 (−0.10 to 0.03)		.28		−.11 (−0.14 to −0.08)		<.001		−.02 (−0.07 to 0.03)			.48							
		Average (mean)	−.03 (−0.07 to −0.00)		.05		−.13 (−0.16 to −0.11)		<.001		−.02 (−0.06 to 0.01)			.17							
		Low (mean − 1 SD)	−.03 (−0.08 to 0.02)		.20		−.15 (−0.19 to −0.11)		<.001		−.03 (−0.07 to 0.02)			.20							
		High versus low	−.00 (−0.10 to 0.09)		.92		.04 (−0.01 to 0.08)		.10		.01 (−0.06 to 0.08)			.77							
	**Physical abuse**																	0.58 (2)			.45
		High (mean + 1 SD)	.01 (−0.07 to 0.09)		.82		−.14 (−0.17 to 0.11)		<.001		−.02 (−0.06 to 0.03)			.49							
		Average (mean)	−.03 (−0.06 to 0.01)		.14		−.12 (−0.14 to −0.09)		<.001		−.02 (−0.06 to 0.01)			.15							
		Low (mean − 1 SD)	−.06 (−0.11 to −0.00)		.03		−.10 (−0.13 to −0.06)		<.001		−.03 (−0.08 to 0.01)			.17							
		High versus low	.07 (−0.05 to 0.18)		.25		−.04 (−0.09 to 0.00)		.06		.02 (−0.05 to −0.08)			.60							
	**Sexual abuse**																	0.01 (2)			.91
		High (mean + 1 SD)	−.03 (−0.09 to 0.03)		.32		−.12 (−0.14 to −0.09)		<.001		−.02 (−0.06 to 0.02)			.34							
		Average (mean)	−.03 (−0.06 to −0.00)		.046		−.13 (−0.15 to −0.10)		<.001		−.02 (−0.06 to 0.01)			.14							
		Low (mean − 1 SD)	−.03 (−0.08 to 0.01)		.15		−.14 (−0.17 to −0.10)		<.001		−.03 (−0.07 to 0.01)			.19							
		High versus low	0 (−0.08 to 0.09)		.93		.02 (−0.01 to 0.05)		.27		.01 (−0.04 to 0.06)			.72							
	**Emotional neglect**																	0.04 (2)			.83
		High (mean + 1 SD)	−.02 (−0.07 to 0.02)		.29		−.11 (−0.14 to −0.08)		<.001		−.02 (−0.07 to 0.03)			.40							
		Average (mean)	−.03 (−0.06 to −0.00)		.04		−.13 (−0.15 to −0.10)		<.001		−.02 (−0.06 to 0.01)			.15							
		Low (mean − 1 SD)	−.04 (−0.08 to 0.00)		.07		−.15 (−0.19 to −0.11)		<.001		−.03 (−0.07 to 0.02)			.22							
		High versus low	.01 (−0.05 to 0.07)		.64		.04 (−0.0 to 0.09)		.11		0 (−0.06 to 0.07)			.89							
	**Physical neglect**																	0.18 (2)			.67
		High (mean + 1 SD)	−.02 (−0.07 to 0.03)		.41		−.11 (−0.14 to.08)		<.001		−.03 (−0.08 to 0.02)			.29							
		Average (mean)	−.03 (−0.06 to 0.00)		.05		−.13 (−0.15 to −0.10)		<.001		−.03 (−0.06 to 0.01)			.13							
		Low (mean − 1 SD)	−.04 (−0.08 to 0.00)		.07		−.14 (−0.18 to −0.10)		<.001		−.02 (−0.07 to −0.02)			.32							
		High versus low	.02 (−0.05 to 0.09)		.60		.03 (−0.02 to 0.08)		.28		−.00 (−0.08 to 0.07)			.94							

^a^Adjusted for age, sex, ethnicity, level of education, and marital status.

^b^Adjusted *β*, standardized regression coefficients (continuous independent variables were standardized [mean 0, SD 1] for interpreting significant three-way interaction terms and examining the difference in associations between high [mean + 1 SD], average [mean], and low [mean − 1 SD] levels of childhood trauma within and across groups [patients, relatives, and controls]).

^c^Family-wise error-corrected *P* values were computed by multiplying the unadjusted *P* value by the total number of tests to adjust significance levels of Wald tests for three-way interactions.

**Table 7:** Effect of self-esteem and psychotic symptoms at t_n-1_ on self-esteem at t_n_ by group and modified by childhood trauma^a^.

			Patients					Relatives					Controls					Wald test for interaction								
			Adjusted *β*^b^ (95% CI)		*P* value		Adjusted *β* (95% CI)			*P* value		Adjusted *β* (95% CI)			*P* value		*χ*^2^ (*df*)				*P* value^c^					
**Psychotic Experiences t_n-1_**																										
	**Emotional abuse**																			0.52 (2)			.47			
		High (mean + 1 SD)		−.09 (−0.22 to 0.05)		.20			−.09 (−0.12 to −0.06)		<.001			−.13 (−0.27 to 0.00)		.06										
		Average (mean)		−.09 (−0.14 to −0.04)		.001			−.11 (−0.14 to −0.08)		<.001			−.08 (−0.15 to −0.02)		.02										
		Low (mean − 1 SD)		−.09 (−0.19 to −0.00)		.046			−.13 (−0.18 to −0.09)		<.001			−.03 (−0.13 to −0.07)		.51										
		High versus low		.01 (−0.20 to 0.21)		.96			.04 (−0.01 to 0.10)		.09			−.10 (−0.29 to −0.10)		.33										
	**Physical abuse**																			0.04 (2)			.84			
		High (mean + 1 SD)		−.07 (−0.21 to 0.07)		.32			−.10 (−0.13 to −0.07)		<.001			−.04 (−0.15 to 0.07)		.46										
		Average (mean)		−.09 (−0.14 to −0.03)		.002			−.10 (−0.13 to −0.08)		<.001			−.07 (−0.13 to −0.01)		.03										
		Low (mean − 1 SD)		−.10 (−0.20 to −0.01)		.03			−.11 (−0.15 to −0.07)		<.001			−.10 (−0.20 to −0.00)		.04										
		High versus low		.03 (−0.17 to 0.24)		.75			−.01 (−0.03 to 0.06)		.48			.06 (−0.11 to 0.22)		.48										
	**Sexual abuse**																			0.02 (2)			.88			
		High (mean + 1 SD)		−.07 (−0.19 to 0.05)		.24			−.10 (−0.13 to −0.07)		<.001			−.06 (−0.15 to −0.02)		.15										
		Average (mean)		−.09 (−0.14 to −0.04)		<.001			−.10 (−0.13 to −0.07)		<.001			−.07 (−0.13 to −0.01)		.03										
		Low (mean − 1 SD)		−.11 (−0.20 to −0.01)		.03			−.10 (−0.14 to −0.07)		<.001			−.08 (−0.16 to 0.00)		.055										
		High versus low		.03 (−0.16 to 0.23)		.72			.00 (−0.04 to 0.04)		.85			.02 (−0.09 to 0.13)		.75										
	**Emotional neglect**																			0.00 (2)			.99			
		High (mean + 1 SD)		−.10 (−0.17 to −0.02)		.01			−.10 (−0.14 to −0.06)		<.001			−.08 (−0.19 to 0.04)		.18										
		Average (mean)		−.09 (−0.14 to −0.04)		<.001			−.10 (−0.13 to −0.08)		<.001			−.07 (−0.14 to −0.01)		.03										
		Low (mean − 1 SD)		−.08 (−0.15 to −0.02)		.01			−.11 (−0.15 to −0.06)		<.001			−.07 (−0.16 to −0.03)		.18										
		High versus low		−.02 (−0.12 to 0.08)		.76			.01 (−0.05 to 0.07)		.83			−.01 (−0.18 to 0.15)		.87										
	**Physical neglect**																			0.48 (2)			.49			
		High (mean + 1 SD)		−.09 (−0.16 to −0.01)		.02			−.10 (−0.13 to −0.07)		<.001			−.00 (−0.19 to 0.18)		.96										
		Average (mean)		−.09 (−0.14 to −0.04)		<.001			−.11 (−0.14 to −0.08)		<.001			−.06 (−0.13 to 0.02)		.12										
		Low (mean − 1 SD)		−.09 (−0.16 to −0.03)		.006			−.12 (−0.16 to −0.07)		<.001			−.11 (−0.22 to 0.00)		.05										
		High versus low		.01 (−0.09 to 0.11)		.89			.02 (−0.03 to 0.07)		.41			−.11 (−0.16 to 0.37)		.43										

^a^Adjusted for age, sex, ethnicity, level of education, and marital status.

^b^Adjusted *β*, standardized regression coefficients (continuous independent variables were standardized [mean 0, SD 1] for interpreting significant three-way interaction terms and examining the difference in associations between high [mean + 1 SD], average [mean], and low [mean−1 SD] levels of childhood trauma within and across groups [patients, relatives, and controls]).

^c^Family-wise error-corrected *P* values were computed by multiplying the unadjusted *P* value by the total number of tests to adjust significance levels of Wald tests for three-way interactions.

## Discussion

### Principal Findings

Using an experience sampling design, the results of this study showed strong evidence that associations between momentary low self-esteem and increased intensity of psychotic experiences in daily life were modified by several types of childhood trauma, that is, physical and sexual abuse and physical neglect. For physical abuse, this was only the case for relatives of patients with psychotic disorders, and for physical neglect, this was only the case for patients. Sexual abuse modified the association in relatives and controls, but not in patients. Emotional abuse and emotional neglect did not modify the association between self-esteem and psychotic experiences in any group. When investigating temporal order, we found no evidence that childhood trauma modified the temporal associations between self-esteem at t_n-1_ and psychotic experiences at t_n_ or those between psychotic experiences at t_n-1_ and self-esteem at t_n_.

### Methodological Considerations

Several limitations of this study should be considered when interpreting its findings. First, only a selection of the baseline sample made it through the third wave. For our analyses, we had to exclude 118 individuals because they did not complete either a sufficient number of ESM assessments or the CTQ. Possibly, these assessments were too burdensome and, therefore, might have led to selection bias. However, when comparing the excluded individuals with those in the analytical sample, who participated in the third wave of GROUP assessments, in terms of basic sample characteristics, the included and excluded participants were comparable to a great extent, except for ethnicity (included participants were more often White in all 3 groups). Second, the CTQ, which was used to measure exposure to several types of trauma during childhood, is a retrospective self-report measure. It has been argued that the CTQ is prone to recall bias and that the manifestation of psychotic symptoms might affect the ratings of this measure [62,63]. However, Gayer-Anderson et al [64] demonstrated the accuracy, strength of agreement, and convergent validity to be broadly similar between patients with first-episode psychosis and controls. Notably, although measured differently, in our control group, the prevalence of several childhood trauma types was higher than that in the general population in the Netherlands [65]. However, if we only look at the categories “severe to extreme” and “moderate to severe” in our sample, the percentages are quite similar, except for sexual abuse. Possibly related to this, we found that lower levels of self-esteem were associated with more intense psychotic experiences in controls exposed to high versus low levels of sexual abuse; this was not the case for any of the other types of childhood trauma (in controls). This might indicate that higher levels of sexual abuse in this group might have affected the outcome. In addition, all ESM assessments of self-esteem and psychotic experiences were based on subjective self-reports, which might have led to bias. However, the ESM has been found to be a feasible, reliable, and valid assessment method in various populations [45,51,66], including patients with psychosis [51,52,58].

Moreover, momentary self-esteem was measured using only 2 items. Using fewer items in ESM research is quite common because it minimizes reactivity to the assessment method [67]. The construct of self-esteem involves both positive and negative self-esteem [68,69]. Therefore, we used 1 item that measured the positive dimension and 1 that measured the negative dimension of self-esteem, which is in line with previous ESM studies investigating self-esteem [55,70]. Because of this heterogeneity of the self-esteem construct and, hence, ESM items, the internal consistency of the 2 momentary self-esteem items that we used was as expected (within-person *α*=.22; between-person *α*=.76) [71]. In previous analyses, we investigated the extent to which the 2 ESM items adequately tapped into the self-esteem construct in a subsample of the current sample. We found that the convergent validity between the 2 momentary self-esteem items and the Rosenberg Self-Esteem Scale [69] was fair, with the intraclass correlation coefficient being 0.41 [33]. It would have been interesting, however, to triangulate momentary ESM measures with explicit (Rosenberg Self-Esteem Scale) and implicit measures [72] of self-esteem to corroborate our findings. In addition, for future research, we would recommend using multiple items to measure both the positive and negative dimensions of momentary self-esteem.

Next, we controlled for potential a priori confounders, such as age, gender, ethnicity, education level, and marital status. We did not include medication use as a confounder because 98% (98/100) of the patients were using medication. Nevertheless, unmeasured confounders, such as other childhood adversities, comorbidities, the impact of illness chronicity, and molecular genetic measures, were not considered and may have, therefore, influenced the findings.

Finally, we standardized the continuous childhood trauma variables because it allowed us to interpret associations at higher versus lower levels of childhood trauma [60,61]. Notably, this implies that by using continuous and standardized variables for childhood trauma, no discrete distinction was made between those exposed and not exposed, rather between those exposed to higher and lower levels of each type of childhood trauma. Therefore, these results could possibly be an underestimation of the reality.

### Comparison With Previous Research

There is well-established evidence that exposure to childhood trauma is a risk factor for developing a psychotic disorder [3-8,10-12], and self-esteem has been proposed to be involved in the pathway from childhood trauma to psychosis [38-42]. In addition, mediation models linking childhood trauma and self-esteem in pathways to psychosis have been previously proposed and tested [41,73]. However, evidence regarding whether prior exposure to childhood trauma (emotional, physical, and sexual abuse and emotional and physical neglect) moderates the association between momentary self-esteem and psychotic experiences in daily life remains limited.

As hypothesized (hypothesis 1), and extending previous findings [33], we found evidence that exposure to physical and sexual abuse and physical neglect modified the association between momentary self-esteem and psychotic experiences in daily life. However, the results also suggested that exposure to emotional abuse and emotional neglect did not modify the association between momentary self-esteem and psychotic experiences in daily life. This is unexpected, as previous studies demonstrated that when comparing the 5 types of childhood trauma, emotional abuse and neglect are the types that affect self-esteem the most [74,75]. Therefore, we would expect that especially these types of trauma would have a particular bearing on the association between self-esteem and psychosis. However, the timing, chronicity, and severity of emotional abuse and neglect are related to the extent to which maltreatment affects developmental trajectories [76]. For example, when maltreatment is less severe or ends early in childhood, it is possible that its impact might fade with time in some individuals [77,78]. Indeed, our data show that the vast majority of the participants experienced none or mild to moderate levels of emotional abuse and neglect. Another explanation for these unexpected results might be that cross-sectional modeling of associations between momentary self-esteem and psychotic experiences does not consider moment-to-moment variation in these measures. However, when we evaluated these associations using time lags, we observed that exposure to childhood trauma did not modify the temporal association between self-esteem and psychotic experiences and vice versa.

On the basis of previous literature [38-42], we hypothesized that the effect of exposure to childhood trauma would modify the association between low self-esteem and increased intensity of psychotic experiences in daily life most substantially in patients, followed by their relatives. We found that physical neglect modified the association between self-esteem and psychotic experiences in patients; physical abuse modified this association only in relatives; and sexual abuse modified this association in relatives and controls, with the impact being stronger in relatives than in controls. It is possible that the controls exposed to childhood trauma may have better coping strategies [79] compared with first-degree relatives of patients with psychotic disorders who have a familial liability to psychosis. Therefore, controls might be more resilient to lower levels of self-esteem and psychotic experiences in daily life [79]. In line with this, there is also evidence that controls exposed to high levels of childhood trauma are more resilient to daily life stress, compared with patients with first-episode psychosis, individuals with at-risk mental state, and help-seeking service users [22,23]. Moreover, previous literature showed that resilience at baseline was lower in individuals who developed psychosis at follow-up than in those who did not [80], suggesting that resilience is a protective factor in the formation of psychosis [81].

In addition, relatives have a familial liability to psychosis [16,18], and they share a degree of exposure to childhood trauma with their siblings with psychotic disorders [20], which may explain the stronger associations in relatives than in controls. Patients were recruited via treating clinicians, which implies that they all received a form of (standard) mental health care, including medication. Treatments such as psychoeducation, cognitive behavioral therapy, and antipsychotic medication have been shown to reduce symptoms and prevent relapse [82-84]. It has been demonstrated that the therapies target distorted beliefs about delusions and hallucinations, thereby decreasing the negative consequences of psychotic symptoms [83-85]. Moreover, the results of a meta-analysis showed that the interpretation of these beliefs is addressed during treatment by considering psychological mechanisms and processes that might contribute to the formation and maintenance of psychotic symptoms, such as emotions, arousal, attachment, interpersonal issues, trauma, and self-esteem [86]. For example, the results of a randomized clinical trial showed that treatment, such as eye movement desensitization and reprocessing and prolonged exposure, in patients with psychotic disorders reduced trauma symptoms and psychotic symptoms [87], also at 6-month follow-up. This potentially implies that the influence of trauma on patients might become less impactful over time owing to the effects of the treatment they had received. Furthermore, 98% (98/100) of patients were using antipsychotic medication. Emotional flattening might be a negative symptom of schizophrenia, but it is also a side effect of medication. Emotional flattening interferes with expressiveness, and this often leads to problems in interpersonal interactions, which in turn leads to more withdrawal from (social) activities [88]. Selective perception and selective memory are important features of low self-esteem, and as a consequence, negative convictions about one’s self are repeatedly confirmed in new situations [89]. However, this will not occur often if patients who are using antipsychotics socially withdraw and therefore experience fewer social interactions. Potentially, directly or indirectly, medication use and the effects of illness chronicity might have a flattening effect on self-esteem. Therapy and medication use might limit the impact of exposure to traumatic experiences on the association between self-esteem and psychotic experiences in daily life for patients with enduring psychotic disorders.

Overall, the cross-sectional findings indicate that it might possibly be relevant to develop and implement early and low-level interventions that directly target self-esteem, especially for individuals exposed to physical and sexual abuse and physical neglect. Thus, the intensity of psychotic experiences in daily life can be reduced. We are currently evaluating the ecological momentary intervention (EMI) “SELFIE” [90]. The next step would be to implement these types of EMIs in routine mental health care.

Previous research has investigated the temporal order of self-esteem and psychotic experiences in daily life and found that self-esteem preceded psychotic symptoms only in controls and psychotic experiences had a temporal effect on self-esteem in patients with psychotic disorders, their first-degree relatives, and controls [33]. In this study, we investigated whether prior exposure to childhood trauma modified the temporal associations between self-esteem at t_n-1_ and psychotic experiences at t_n_ and between psychotic experiences at t_n-1_ and self-esteem at t_n_. However, the results showed no evidence of these associations. Nevertheless, we would not rule out the hypothesis of a temporal order entirely because, in this study, we investigated the interaction between prior exposure to childhood trauma, psychotic experiences at t_n_, and momentary self-esteem at the previous time point (t_n-1_) and vice versa. Assessments took place within 90-minute time blocks. It is possible that longer time lags would have yielded different results, as the occurrence of psychosis might be preceded by weeks, months, or even years of psychological and behavioral abnormalities [91]. Perhaps self-esteem is a process that needs more time to unfold to be succeeded by psychotic experiences.

### Conclusions

Taken together, our findings suggest that the association between self-esteem and psychotic experiences in daily life is stronger in those exposed to high versus low levels of several types of childhood trauma. Hence, self-esteem might be a psychological process that links childhood trauma to psychosis. The results showed that this is especially the case in first-degree relatives and, to a lesser extent, in patients with psychotic disorders. Although we did not find evidence of temporal associations, the cross-sectional results indicate that improving self-esteem may potentially reduce the intensity of psychotic experiences in daily life. This underlines the importance of developing and evaluating early and low-level EMIs that directly target self-esteem to reduce the intensity of psychotic experiences in daily life. We currently evaluate such an intervention in the SELFIE study [90].

## Appendix

Multimedia Appendix 1 Basic characteristics of the excluded participants (participants with less than one-third experience sampling method assessments or no Childhood Trauma Questionnaire results were excluded).

Multimedia Appendix 2 Associations between self-esteem psychotic experiences at high (mean + 1 SD) versus low (mean − 1 SD) levels of physical abuse, sexual abuse, and physical neglect in patients, relatives, and controls.

## Abbreviations

CTQ: Childhood Trauma Questionnaire
EMI: ecological momentary intervention
ESM: experience sampling method
GROUP: Genetic Risk and Outcome in Psychosis

## References

[1] Duhig M, Patterson S, Connell M, Foley S, Capra C, Dark F, Gordon A, Singh S, Hides L, McGrath JJ, Scott J (2015). The prevalence and correlates of childhood trauma in patients with early psychosis. Aust N Z J Psychiatry.

[2] Bernstein DP, Stein JA, Newcomb MD, Walker E, Pogge D, Ahluvalia T, Stokes J, Handelsman L, Medrano M, Desmond D, Zule W (2003). Development and validation of a brief screening version of the childhood trauma questionnaire. Child Abuse Negl.

[3] Varese F, Smeets F, Drukker M, Lieverse R, Lataster T, Viechtbauer W, Read J, van Os J, Bentall RP (2012). Childhood adversities increase the risk of psychosis: a meta-analysis of patient-control, prospective- and cross-sectional cohort studies. Schizophr Bull.

[4] B Folk J, Tully L, Blacker D, Liles B, Bolden K, Tryon V, Botello R, Niendam T (2019). Uncharted waters: treating trauma symptoms in the context of early psychosis. J Clin Med.

[5] Stanton KJ, Denietolis B, Goodwin BJ, Dvir Y (2020). Childhood trauma and psychosis: an updated review. Child Adolesc Psychiatr Clin N Am.

[6] Frissen A, Lieverse R, Marcelis M, Drukker M, Delespaul P, GROUP Investigators (2015). Psychotic disorder and educational achievement: a family-based analysis. Soc Psychiatry Psychiatr Epidemiol.

[7] Kraan T, van Dam DS, Velthorst E, de Ruigh EL, Nieman DH, Durston S, Schothorst P, van der Gaag M, de Haan L (2015). Childhood trauma and clinical outcome in patients at ultra-high risk of transition to psychosis. Schizophr Res.

[8] Kraan TC, Ising HK, Fokkema M, Velthorst E, van den Berg DP, Kerkhoven M, Veling W, Smit F, Linszen DH, Nieman DH, Wunderink L, Boonstra N, Klaassen RM, Dragt S, Rietdijk J, de Haan L, van der Gaag M (2017). The effect of childhood adversity on 4-year outcome in individuals at ultra high risk for psychosis in the Dutch Early Detection Intervention Evaluation (EDIE-NL) Trial. Psychiatry Res.

[9] Norman RE, Byambaa M, De R, Butchart A, Scott J, Vos T (2012). The long-term health consequences of child physical abuse, emotional abuse, and neglect: a systematic review and meta-analysis. PLoS Med.

[10] Frissen A, Lieverse R, Drukker M, van Winkel R, Delespaul P, GROUP Investigators (2015). Childhood trauma and childhood urbanicity in relation to psychotic disorder. Soc Psychiatry Psychiatr Epidemiol.

[11] Matheson SL, Shepherd AM, Pinchbeck RM, Laurens KR, Carr VJ (2012). Childhood adversity in schizophrenia: a systematic meta-analysis. Psychol Med.

[12] Kraan T, Velthorst E, Smit F, de Haan L, van der Gaag M (2015). Trauma and recent life events in individuals at ultra high risk for psychosis: review and meta-analysis. Schizophr Res.

[13] Bendall S, Hulbert CA, Alvarez-Jimenez M, Allott K, McGorry PD, Jackson HJ (2013). Testing a model of the relationship between childhood sexual abuse and psychosis in a first-episode psychosis group: the role of hallucinations and delusions, posttraumatic intrusions, and selective attention. J Nerv Ment Dis.

[14] Kelleher I, Keeley H, Corcoran P, Ramsay H, Wasserman C, Carli V, Sarchiapone M, Hoven C, Wasserman D, Cannon M (2013). Childhood trauma and psychosis in a prospective cohort study: cause, effect, and directionality. Am J Psychiatry.

[15] Trotta A, Murray RM, Fisher HL (2015). The impact of childhood adversity on the persistence of psychotic symptoms: a systematic review and meta-analysis. Psychol Med.

[16] Guloksuz S, Pries L, Delespaul P, Kenis G, Luykx JJ, Lin BD, Richards AL, Akdede B, Binbay T, Altınyazar V, Yalınçetin B, Gümüş-Akay G, Cihan B, Soygür H, Ulaş H, Cankurtaran E, Kaymak SU, Mihaljevic MM, Petrovic SA, Mirjanic T, Bernardo M, Cabrera B, Bobes J, Saiz PA, García-Portilla MP, Sanjuan J, Aguilar EJ, Santos JL, Jiménez-López E, Arrojo M, Carracedo A, López G, González-Peñas J, Parellada M, Maric NP, Atbaşog Lu C, Ucok A, Alptekin K, Saka MC, Arango C, O'Donovan M, Rutten BP, van Os J, Genetic RiskOutcome of Psychosis (GROUP) investigators (2019). Examining the independent and joint effects of molecular genetic liability and environmental exposures in schizophrenia: results from the EUGEI study. World Psychiatry.

[17] van Os J, van der Steen Y, Islam MA, Gülöksüz S, Rutten BP, Simons CJ (2017). Evidence that polygenic risk for psychotic disorder is expressed in the domain of neurodevelopment, emotion regulation and attribution of salience. Psychol Med.

[18] Islam MA, Khan MF, Quee PJ, Snieder H, van den Heuvel ER, Bruggeman R, Alizadeh BZ, GROUP Investigators (2017). Familial liability to psychosis is a risk factor for multimorbidity in people with psychotic disorders and their unaffected siblings. Eur Psychiatry.

[19] Linscott RJ, van Os J (2012). An updated and conservative systematic review and meta-analysis of epidemiological evidence on psychotic experiences in children and adults: on the pathway from proneness to persistence to dimensional expression across mental disorders. Psychol Med.

[20] Heins M, Simons C, Lataster T, Pfeifer S, Versmissen D, Lardinois M, Marcelis M, Delespaul P, Krabbendam L, van Os J, Myin-Germeys I (2011). Childhood trauma and psychosis: a case-control and case-sibling comparison across different levels of genetic liability, psychopathology, and type of trauma. Am J Psychiatry.

[21] Collip D, Nicolson NA, Lardinois M, Lataster T, van Os J, Myin-Germeys I (2011). Daily cortisol, stress reactivity and psychotic experiences in individuals at above average genetic risk for psychosis. Psychol Med.

[22] Rauschenberg C, van Os J, Cremers D, Goedhart M, Schieveld JN, Reininghaus U (2017). Stress sensitivity as a putative mechanism linking childhood trauma and psychopathology in youth's daily life. Acta Psychiatr Scand.

[23] Reininghaus U, Gayer-Anderson C, Valmaggia L, Kempton MJ, Calem M, Onyejiaka A, Hubbard K, Dazzan P, Beards S, Fisher HL, Mills JG, McGuire P, Craig TK, Garety P, van Os J, Murray RM, Wykes T, Myin-Germeys I, Morgan C (2016). Psychological processes underlying the association between childhood trauma and psychosis in daily life: an experience sampling study. Psychol Med.

[24] Williams J, Bucci S, Berry K, Varese F (2018). Psychological mediators of the association between childhood adversities and psychosis: a systematic review. Clin Psychol Rev.

[25] Krabbendam L, Janssen I, Bak M, Bijl RV, de Graaf R, van Os J (2002). Neuroticism and low self-esteem as risk factors for psychosis. Soc Psychiatry Psychiatr Epidemiol.

[26] Ciufolini S, Morgan C, Morgan K, Fearon P, Boydell J, Hutchinson G, Demjaha A, Girardi P, Doody GA, Jones PB, Murray R, Dazzan P (2015). Self esteem and self agency in first episode psychosis: ethnic variation and relationship with clinical presentation. Psychiatry Res.

[27] Lecomte T, Leclerc C, Wykes T (2018). Symptom fluctuations, self-esteem, and cohesion during group cognitive behaviour therapy for early psychosis. Psychol Psychother.

[28] Smith B, Fowler DG, Freeman D, Bebbington P, Bashforth H, Garety P, Dunn G, Kuipers E (2006). Emotion and psychosis: links between depression, self-esteem, negative schematic beliefs and delusions and hallucinations. Schizophr Res.

[29] Garety PA, Kuipers E, Fowler D, Freeman D, Bebbington PE (2001). A cognitive model of the positive symptoms of psychosis. Psychol Med.

[30] Bentall RP, Kinderman P, Kaney S (1994). The self, attributional processes and abnormal beliefs: towards a model of persecutory delusions. Behav Res Ther.

[31] Kinderman P, Bentall RP (1996). Self-discrepancies and persecutory delusions: evidence for a model of paranoid ideation. J Abnormal Psychol.

[32] Kesting M, Bredenpohl M, Klenke J, Westermann S, Lincoln TM (2013). The impact of social stress on self-esteem and paranoid ideation. J Behav Ther Exp Psychiatry.

[33] Daemen M, van Amelsvoort T, Reininghaus U, Group Investigators (2022). Self-esteem and psychosis in daily life: an experience sampling study. J Psychopathol Clin Sci.

[34] Mansueto G, van Nierop M, Schruers K, Alizadeh BZ, Bartels-Velthuis AA, van Beveren NJ, Bruggeman R, Cahn W, de Haan L, Delespaul P, Meijer CJ, Myin-Germeys I, Kahn RS, Schirmbeck F, Simons CJ, van Haren NE, van Os J, van Winkel R, GROUP Investigators (2018). The role of cognitive functioning in the relationship between childhood trauma and a mixed phenotype of affective-anxious-psychotic symptoms in psychotic disorders. Schizophr Res.

[35] Beck AT (2008). The evolution of the cognitive model of depression and its neurobiological correlates. Am J Psychiatry.

[36] GARETY PA, BEBBINGTON P, FOWLER D, FREEMAN D, KUIPERS E (2007). Implications for neurobiological research of cognitive models of psychosis: a theoretical paper. Psychol Med.

[37] Morgan C, Gayer-Anderson C (2016). Childhood adversities and psychosis: evidence, challenges, implications. World Psychiatry.

[38] Fisher HL, Schreier A, Zammit S, Maughan B, Munafò MR, Lewis G, Wolke D (2013). Pathways between childhood victimization and psychosis-like symptoms in the ALSPAC birth cohort. Schizophr Bull.

[39] Morgan C, Reininghaus U, Fearon P, Hutchinson G, Morgan K, Dazzan P, Boydell J, Kirkbride JB, Doody GA, Jones PB, Murray RM, Craig T (2014). Modelling the interplay between childhood and adult adversity in pathways to psychosis: initial evidence from the AESOP study. Psychol Med.

[40] Gracie A, Freeman D, Green S, Garety PA, Kuipers E, Hardy A, Ray K, Dunn G, Bebbington P, Fowler D (2007). The association between traumatic experience, paranoia and hallucinations: a test of the predictions of psychological models. Acta Psychiatr Scand.

[41] Fisher HL, Appiah-Kusi E, Grant C (2012). Anxiety and negative self-schemas mediate the association between childhood maltreatment and paranoia. Psychiatry Res.

[42] Evans GJ, Reid G, Preston P, Palmier-Claus J, Sellwood W (2015). Trauma and psychosis: the mediating role of self-concept clarity and dissociation. Psychiatry Res.

[43] Birchwood M, Iqbal Z (1998). Depression and suicidal thinking in psychosis: a cognitive approach. Outcome and Innovation in Psychological Treatment of Schizophrenia.

[44] Iqbal Z, Birchwood M, Chadwick P, Trower P (2018). Cognitive approach to depression and suicidal thinking in psychosis. Br J Psychiatry.

[45] Csikszentmihalyi M, Larson R (1987). Validity and reliability of the experience-sampling method. J Nerv Ment Dis.

[46] Cardno AG, Marshall EJ, Coid B, Macdonald AM, Ribchester TR, Davies NJ, Venturi P, Jones LA, Lewis SW, Sham PC, Gottesman II, Farmer AE, McGuffin P, Reveley AM, Murray RM (1999). Heritability estimates for psychotic disorders: the Maudsley twin psychosis series. Arch Gen Psychiatry.

[47] Korver N, Quee PJ, Boos HB, Simons CJ, de Haan L, GROUP investigators (2012). Genetic Risk and Outcome of Psychosis (GROUP), a multi-site longitudinal cohort study focused on gene-environment interaction: objectives, sample characteristics, recruitment and assessment methods. Int J Methods Psychiatr Res.

[48] (1994). Diagnostic and Statistical Manual of Mental Disorders, 4th edition (DSM-IV).

[49] Delespaul P (1995). Assessing Schizophrenia in Daily Life : The Experience Sampling Method.

[50] Standaard Onderwijsindeling 2006. Centraal Bureau voor de Statistiek.

[51] Myin-Germeys I, Oorschot M, Collip D, Lataster J, Delespaul P, van Os J (2009). Experience sampling research in psychopathology: opening the black box of daily life. Psychol Med.

[52] Palmier-Claus JE, Dunn G, Lewis SW (2011). Emotional and symptomatic reactivity to stress in individuals at ultra-high risk of developing psychosis. Psychol Med.

[53] Myin-Germeys I, van Os J, Schwartz JE, Stone AA, Delespaul PA (2001). Emotional reactivity to daily life stress in psychosis. Arch Gen Psychiatry.

[54] Palmier-Claus JE, Myin-Germeys I, Barkus E, Bentley L, Udachina A, Delespaul PA, Lewis SW, Dunn G (2011). Experience sampling research in individuals with mental illness: reflections and guidance. Acta Psychiatr Scand.

[55] Hermans K, van der Steen Y, Kasanova Z, van Winkel R, Reininghaus U, Lataster T, Bechdolf A, Gimpel-Drees J, Wagner M, Myin-Germeys I (2020). Temporal dynamics of suspiciousness and hallucinations in clinical high risk and first episode psychosis. Psychiatry Res.

[56] Geldhof GJ, Preacher KJ, Zyphur MJ (2014). Reliability estimation in a multilevel confirmatory factor analysis framework. Psychol Methods.

[57] Oorschot M, Kwapil T, Delespaul P, Myin-Germeys I (2009). Momentary assessment research in psychosis. Psychol Assess.

[58] Myin-Germeys I, Delespaul P, van Os J (2005). Behavioural sensitization to daily life stress in psychosis. Psychol Med.

[59] Aiken L, West S, Reno R (1991). Multiple Regression Testing and Interpreting Interactions.

[60] Cohen J, Cohen P, West S, Aiken L (1975). Applied Multiple Regression/Correlation Analysis for the Behavioral Sciences.

[61] Dawson JF, Richter AW (2006). Probing three-way interactions in moderated multiple regression: development and application of a slope difference test. J Appl Psychol.

[62] Fisher HL, Craig TK, Fearon P, Morgan K, Dazzan P, Lappin J, Hutchinson G, Doody GA, Jones PB, McGuffin P, Murray RM, Leff J, Morgan C (2011). Reliability and comparability of psychosis patients' retrospective reports of childhood abuse. Schizophr Bull.

[63] Maughan B, Rutter M (1997). Retrospective reporting of childhood adversity: issues in assessing long-term recall. J Pers Disord.

[64] Gayer-Anderson C, Reininghaus U, Paetzold I, Hubbard K, Beards S, Mondelli V, Di Forti M, Murray RM, Pariante CM, Dazzan P, Craig TJ, Fisher HL, Morgan C (2020). A comparison between self-report and interviewer-rated retrospective reports of childhood abuse among individuals with first-episode psychosis and population-based controls. J Psychiatr Res.

[65] Euser S, Alink LR, Pannebakker F, Vogels T, Bakermans-Kranenburg MJ, Van IJzendoorn MH (2013). The prevalence of child maltreatment in the Netherlands across a 5-year period. Child Abuse Negl.

[66] Reininghaus U, Depp CA, Myin-Germeys I (2016). Ecological interventionist causal models in psychosis: targeting psychological mechanisms in daily life. Schizophr Bull.

[67] Gabriel AS, Podsakoff NP, Beal DJ, Scott BA, Sonnentag S, Trougakos JP, Butts MM (2018). Experience sampling methods: a discussion of critical trends and considerations for scholarly advancement. Organiz Res Method.

[68] Udachina A, Bentall RP, Varese F, Rowse G (2017). Stress sensitivity in paranoia: poor-me paranoia protects against the unpleasant effects of social stress. Psychol Med.

[69] Rosenberg M (1965). Society and the Adolescent Self-Image.

[70] Palmier-Claus JE, Dunn G, Morrison AP, Lewis SW (2011). The role of metacognitive beliefs in stress sensitisation, self-esteem variability, and the generation of paranoia. Cogn Neuropsychiatry.

[71] Udachina A, Thewissen V, Myin-Germeys I, Fitzpatrick S, O'kane A, Bentall RP (2009). Understanding the relationships between self-esteem, experiential avoidance, and paranoia: structural equation modelling and experience sampling studies. J Nerv Ment Dis.

[72] Greenwald AG, McGhee DE, Schwartz JL (1998). Measuring individual differences in implicit cognition: the implicit association test. J Personal Social Psychol.

[73] Bebbington P, Jonas S, Kuipers E, King M, Cooper C, Brugha T, Meltzer H, McManus S, Jenkins R (2011). Childhood sexual abuse and psychosis: data from a cross-sectional national psychiatric survey in England. Br J Psychiatry.

[74] Briere J, Runtz M (1990). Differential adult symptomatology associated with three types of child abuse histories. Child Abuse Negl.

[75] Finzi-Dottan R, Karu T (2006). From emotional abuse in childhood to psychopathology in adulthood: a path mediated by immature defense mechanisms and self-esteem. J Nerv Ment Dis.

[76] Toth SL, Cicchetti D (2013). A developmental psychopathology perspective on child maltreatment. Introduction. Child Maltreat.

[77] Thornberry TP, Ireland TO, Smith CA (2002). The importance of timing: the varying impact of childhood and adolescent maltreatment onmultiple problem outcomes. Dev Psychopathol.

[78] Iwaniec D, Larkin E, Higgins S (2006). Research review: risk and resilience in cases of emotional abuse. Child Fam Soc Work.

[79] Villasana M, Alonso-Tapia J, Ruiz M (2016). A model for assessing coping and its relation to resilience in adolescence from the perspective of “person–situation interaction”. Personal Indiv Differ.

[80] Kim KR, Song YY, Park JY, Lee EH, Lee M, Lee SY, Kang JI, Lee E, Yoo SW, An SK, Kwon JS (2013). The relationship between psychosocial functioning and resilience and negative symptoms in individuals at ultra-high risk for psychosis. Aust N Z J Psychiatry.

[81] Patel V, Goodman A (2007). Researching protective and promotive factors in mental health. Int J Epidemiol.

[82] Lincoln TM, Wilhelm K, Nestoriuc Y (2007). Effectiveness of psychoeducation for relapse, symptoms, knowledge, adherence and functioning in psychotic disorders: a meta-analysis. Schizophr Res.

[83] Hutton P, Taylor PJ (2013). Cognitive behavioural therapy for psychosis prevention: a systematic review and meta-analysis. Psychol Med.

[84] Crossley NA, Constante M, McGuire P, Power P (2010). Efficacy of atypical v. typical antipsychotics in the treatment of early psychosis: meta-analysis. Br J Psychiatry.

[85] Butler AC, Chapman JE, Forman EM, Beck AT (2006). The empirical status of cognitive-behavioral therapy: a review of meta-analyses. Clin Psychol Rev.

[86] Sitko K, Bewick BM, Owens D, Masterson C (2020). Meta-analysis and meta-regression of cognitive behavioral therapy for psychosis (CBTp) across time: the effectiveness of CBTp has improved for delusions. Schizophrenia Bull Open.

[87] van den Berg DP, de Bont PA, van der Vleugel BM, de Roos C, de Jongh A, Van Minnen A, van der Gaag M (2015). Prolonged exposure vs eye movement desensitization and reprocessing vs waiting list for posttraumatic stress disorder in patients with a psychotic disorder: a randomized clinical trial. JAMA Psychiatry.

[88] Haverkampf CJ (2013). Antipsychotics: emotional flattening vs apathy. J Psychiatry Psychother Commun.

[89] de Neef M (2015). Build Your Confidence with CBT 6 Simple Steps to Be Happier, More Successful and Fulfilled.

[90] Daemen M, Postma MR, Lindauer R, Hoes-van der Meulen I, Nieman D, Delespaul P, Breedvelt JJ, van der Gaag M, Viechtbauer W, Schruers K, van den Berg D, Bockting C, van Amelsvoort T, Reininghaus U (2021). Efficacy of a transdiagnostic ecological momentary intervention for improving self-esteem (SELFIE) in youth exposed to childhood adversity: study protocol for a multi-center randomized controlled trial. Trials.

[91] Larson MK, Walker EF, Compton MT (2010). Early signs, diagnosis and therapeutics of the prodromal phase of schizophrenia and related psychotic disorders. Expert Rev Neurother.

