# Supplementary material for: Momentary Self-esteem as a Process Underlying the Association Between Childhood Trauma and Psychosis: Experience Sampling Study
Source: JMIR Ment Health. 2023 Apr 5;10:e34147. doi: 10.2196/34147 (PMC10131675; doi:10.2196/34147)
Supplement: Multimedia Appendix 1 [file mental_v10i1e34147_app1.docx]

**Supplementary Table S1**. Basic Characteristics of the excluded participants (participants with less than one third ESM assessments and no CTQ results were excluded)

|  | **Excluded**  **patients**  **(n=55)** | **Compared to  incl. patients** | |  | **Excluded**  **relatives**  **(n=51)** | **Compared to**  **incl. relatives** | |  | **Excluded**  **controls**  **(n=12)** | **Compared to**  **incl. controls** | |
| --- | --- | --- | --- | --- | --- | --- | --- | --- | --- | --- | --- |
|  |  | **Test statistics** | **p** |  |  | **Test statistics** | **P** |  |  | **Test statistics** | **P** |
| **Age (years), mean (SD)** | 32.6 (5.5) | z=1.07 | 0.284 |  | 32.6 (8.2) | z=1.95 | 0.051 |  | 33.5 (9.5) | z=1.95 | 0.051 |
| **Gender, n (%)**  **Men**  **Women** | 41 (74.5)  14 (25.5) | $\chi$^2^=1.49, df=1 | 0.222 |  | 20 (39.2)  31 (60.8) | $\chi$^2^=0.01, df=1 | 0.940 |  | 5 (41.7)  7 (58.3) | $\chi$^2^=0.72, df=1 | 0.395 |
| **Ethnicity, n (%)^a^**  **Caucasian**  **Non-caucasian** | 38 (71.7)  15 (28.3) | $\chi$^2^=7.99, df=1 | 0.005 |  | 37 (72.6)  13 (27.4) | $\chi$^2^=7.98, df=1 | 0.005 |  | 10 (83.3)  2 (16.7) | $\chi$^2^=3.98, df=1 | 0.046 |
| **Level of education, n (%)^b^**  **Low**  **Middle**  **High** | 1 (8.3)  8 (66.7)  3 (25.0) | $\chi$^2^=2.83, df=2 | 0.243 |  | 1 (7.1)  4 (28.6)  9 (64.3) | $\chi$^2^=0.50, df=2 | 0.779 |  | 0 (0.0)  1 (25.0)  3 (75.0) | $\chi$^2^=0.43, df=2 | 0.805 |
| **Marital status, n (%)**  **Not married**  **Married/live together**  **Divorced** | 44 (80.0)  10 (18.2)  1 (1.8) | $\chi$^2^=2.37, df=2 | 0.306 |  | 22 (43.1)  27 (53.0)  2 (3.9) | $\chi$^2^=2.19, df=2 | 0.334 |  | 3 (25.0)  9 (75.0)  0 (0) | $\chi$^2^=0.90, df=2 | 0.638 |
| **Current antipsychotic use, n ^c^** | 40 (100.0) | $\chi$^2^= 1.29, df=3 | 0.731 |  | - |  |  |  | - |  |  |

Missing values (%): ^a^ 2.5, ^b^ 74.6, ^c^ 12.7. ESM, experience sampling method, CTQ, childhood trauma questionnaire.
