# Supplementary material for: Momentary Self-esteem as a Process Underlying the Association Between Childhood Trauma and Psychosis: Experience Sampling Study
Source: JMIR Ment Health. 2023 Apr 5;10:e34147. doi: 10.2196/34147 (PMC10131675; doi:10.2196/34147)
Supplement: Multimedia Appendix 2 [file mental_v10i1e34147_app2.docx]

**Multimedia Appendix 2**

**Figure S1.** Association between self-esteem at high (mean+1 SD) and low (mean-1 SD) levels of physical abuse in patients, relatives and controls.

Patients Relatives Controls

*Note:* High_trauma defined as 1 SD above the mean level of physical abuse; Low_trauma defined as 1 SD below the mean level of physical abuse. Adjusted for age, gender, ethnicity, level of education, and marital status.

**Figure S2.** Association between self-esteem at high (mean+1 SD) and low (mean-1 SD) levels of sexual abuse in patients, relatives and controls.

 Patients Relatives Controls

*Note:* High_trauma defined as 1 SD above the mean level of sexual abuse; Low_trauma defined as 1 SD below the mean level of sexual abuse. Adjusted for age, gender, ethnicity, level of education, and marital status.

**Figure S3**. Association between self-esteem at high (mean+1 SD) and low (mean-1 SD) levels of physical neglect in patients, relatives and controls.

Patients Relatives Controls

*Note:* High_trauma defined as 1 SD above the mean level of physical neglect; Low_trauma defined as 1 SD below the mean level of physical neglect. Adjusted for age, gender, ethnicity, level of education, and marital status.
